# Sdy-1 Executes Antitumor Activity in HepG2 and HeLa Cancer Cells by Inhibiting the Wnt/β-Catenin Signaling Pathway

**DOI:** 10.3390/md20020125

**Published:** 2022-02-05

**Authors:** Mengyu Sun, Dongdong Zhou, Jingwan Wu, Jing Zhou, Jing Xu

**Affiliations:** 1 One Health Institute, School of Chemical Engineering and Technology, Hainan University, Haikou 570228, China; summer@hainanu.edu.cn (M.S.); dongdongchoy@hainanu.edu.cn (D.Z.); 2021110817000019@hainanu.edu.cn (J.W.); 2 Hainan Provincial Fine Chemical Engineering Research Center, School of Life Sciences, Hainan University, Haikou 570228, China; 993725@hainanu.edu.cn

**Keywords:** mangrove endophytic *Pestalotiopsis*, Sdy-1, antitumor activity, Wnt signaling, β-catenin

## Abstract

Demethylincisterol A_3_ (Sdy-1), a highly degraded sterol that we previously isolated from Chinese mangrove *Rhizophora mucronata* endophytic *Pestalotiopsis* sp. HQD-6, exhibits potent antitumor activity towards a variety of cancer cells. In this study, we further verified that Sdy-1 effectively inhibited the proliferation and migration of human liver (HepG2) and cervical cancer (HeLa) cells in vitro and it can induce cell apoptosis and arrest the cell cycle in the G1-phase. Mechanistically, we demonstrated that Sdy-1 executes its function via inhibition of the Wnt/β-catenin signaling pathway. Sdy-1 may not inhibit the Wnt signaling pathway through the cascade reaction from upstream to downstream, but directly acts on β-catenin to reduce its transcription level, thereby reducing the level of β-catenin protein and further reducing the expression of downstream related proteins. The possible interaction between Sdy-1 and β-catenin protein was further confirmed by molecular docking studies. In the nude mouse xenograft model, Sdy-1 can also significantly inhibit tumor growth. These results indicated that Sdy-1 is an efficient inhibitor of the Wnt signaling pathway and is a promising antitumor candidate for therapeutic applications.

## 1. Introduction

The Wnt signaling pathway plays an essential role in various cellular responses and oncogenesis, including cell morphology, proliferation, motility, and differentiation [1,2]. β-catenin is a key component of the Wnt signaling pathway and acts as a coactivator for transcription factors of the T-cell factor / lymphoid- enhancing (TCF/LEF) family [3], and the bulk of β-catenin content in the cell is modulated by β-catenin protein stabilization. A complex of scaffolding protein Axin, glycogen synthase kinase-3β (GSK3β), and adenomatous polyposis coli (APC) protein in the cytoplasm (Axin/GSK3β/APC) catalyzes β-catenin phosphorylation and sequentially leads to its recognition of ubiquitin-dependent proteasomal degradation in normal cells. The Wnt/β-catenin signaling is activated when the Wnt protein combines with the frizzled (fzd) receptor and the low-density lipoprotein receptor-related protein 5/6 (LRP5/6), which together recruit the scaffolding protein Dishevelled (Dvl). This results in a consecutive inhibition of GSK3β [4,5]. The recruited Axin complex induces unphosphorylated β-catenin, which is stabilized in the cytoplasm to translocate into the nucleus where it forms complexes with T-cell transcription factor (TCF)/lymphoid enhancer-binding factor (LEF). These complexes activate transcription of Wnt downstream regulatory genes, including Cyclin D1, CDK4, c-myc, and related genes [6].

Liver cancer is one of the six most common cancers in the world, with the seventh-highest incidence of cancer and the third-highest mortality rate, it is the leading cause of cancer death among men under 60 in China [7,8,9]. Primary liver cancer comprises hepatocellular carcinoma (HCC), intrahepatic cholangiocarcinoma (iCCA), and other rare tumors, notably fibrolamellar carcinoma and hepatoblastoma [10]. Although hepatoblastoma accounts for a small proportion of liver malignancies, it is the most common liver malignancy in children, and in recent years its incidence has increased slowly and steadily at a rate of 1.2 to 1.5 new cases per million people per year [11,12]. At present, the treatment of hepatoblastoma is mainly chemotherapy and surgical resection, but the prognosis is still not optimistic [13]. Therefore, there is an urgent need to find personalized targeted therapies [14]. CTNNB1 (encoding β-catenin), APC and Axin mutations can be detected in up to 80% of cases of hepatoblastoma, indicating that the Wnt/β-catenin signaling pathway plays a vital role in the formation of hepatoblastoma [15,16,17]. Although cervical cancer is preventable, it remains the fourth most common cancer in females. Approximately 528,000 females are diagnosed with cervical cancer each year worldwide. Cervical cancer results in 266,000 deaths per year, of which 85% occur in developing countries [18]. Cervical cancer has no obvious symptoms at an early stage; therefore, most cases have reached an advanced stage when detected [19,20]. Shinohara et al. reported that β-catenin expression was increased in 73% of cervical cancer samples, with positive nuclear and cytoplasmic staining, and gene mutations were present in 20% of cases [21]. Thus, the use of Wnt pathway antagonists may be a potential therapeutic strategy for the treatment of cervical carcinogenesis.

Mangrove endophytic fungi are considered to be a promising source of structurally unique and biologically active natural products, so they are receiving increasing attention in academia and industries [22,23]. One fungal genus, *Pestalotiopsis*, is especially known for producing a wide variety of biologically active compounds [24,25,26,27]. Our preliminary screening for in vitro cytotoxicity showed that an endogenous strain of *Pestalotiopsis* sp. HQD-6 has a strong inhibitory effect on the growth of some human cancer cell lines (e.g., HeLa and HepG2 cells) with IC_50_ values of 13.01 ± 1.91 μg/mL and 9.58 ± 0.01 μg/mL, respectively. Chemical investigation of HQD-6 led to the isolation of a highly degraded sterol, demethylincisterol A_3_ (Sdy-1), its structure was unequivocally determined by extensive NMR spectroscopic experiments as well as mass spectrometry (Figures S1–S8) and comparison with data reported in the literature [28,29]. It was first reported as a synthetic intermediate in the synthesis of 17-methylincisterol and was isolated from different kinds of edible and medicinal mushrooms and marine sponges [29,30,31]. This compound has cytotoxic effects on certain tumor cell lines [32]. In earlier studies in our laboratory, Sdy-1 was found to be highly cytotoxic to HepG2 and HeLa cells with IC_50_ values reached nM degree [28], but its specific mechanism is still unclear. Therefore, we studied the specific mechanism of action of Sdy-1 and found evidence that Sdy-1 reduces the viability of HepG2 and HeLa cells by inhibiting the Wnt signaling pathway.

## 2. Results

### 2.1. Sdy-1 Inhibits HepG2 and HeLa Cell Proliferation In Vitro

**Figure 1:**
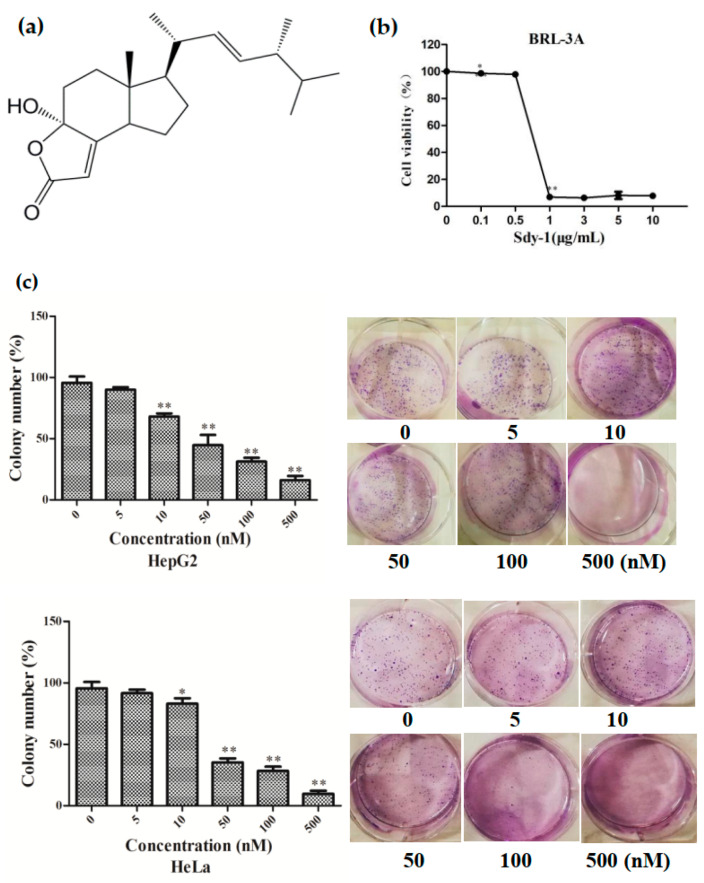
Effects of Sdy-1 on the growth of cells. (**a**) Chemical structure of Sdy-1. (**b**) Cytotoxic effects of Sdy-1 on normal rat liver cell. (**c**) Effect of Sdy-1 on clone formation. Data are expressed as a mean ± SD of three independent tests. * *p* < 0.05, ** *p* < 0.01 compared to the control group.

The chemical structure of Sdy-1 is shown in Figure 1a. We have proven in our previous research that Sdy-1 significantly hinders the proliferation of HepG2 and HeLa cells in vitro. The IC_50_ values of Sdy-1 for HepG2 and HeLa cells were 14.16 ± 0.56 and 0.17 ± 0.00 nM, respectively [28], while the IC_50_ of Sdy-1 to normal rat liver cell was 1.58 ± 0.05 μm (Figure 1b), which indicates that Sdy-1 has a selective killing effect. We further evaluated the long-term inhibitory effect of Sdy-1 on these two kinds of cells by colony formation assays. These assays showed that Sdy-1 had a noteworthy inhibitory impact on the colony-forming ability of HepG2 and HeLa tumor cells. The higher the Sdy-1 concentration, the lower the colony formation rate, which indicates that Sdy-1 has a strong long-term inhibitory effect on HepG2 and HeLa cell growth (Figure 1c).

### 2.2. Sdy-1 Inhibits Migration and Invasion Progress of HepG2 and HeLa Cells 

**Figure 2:**
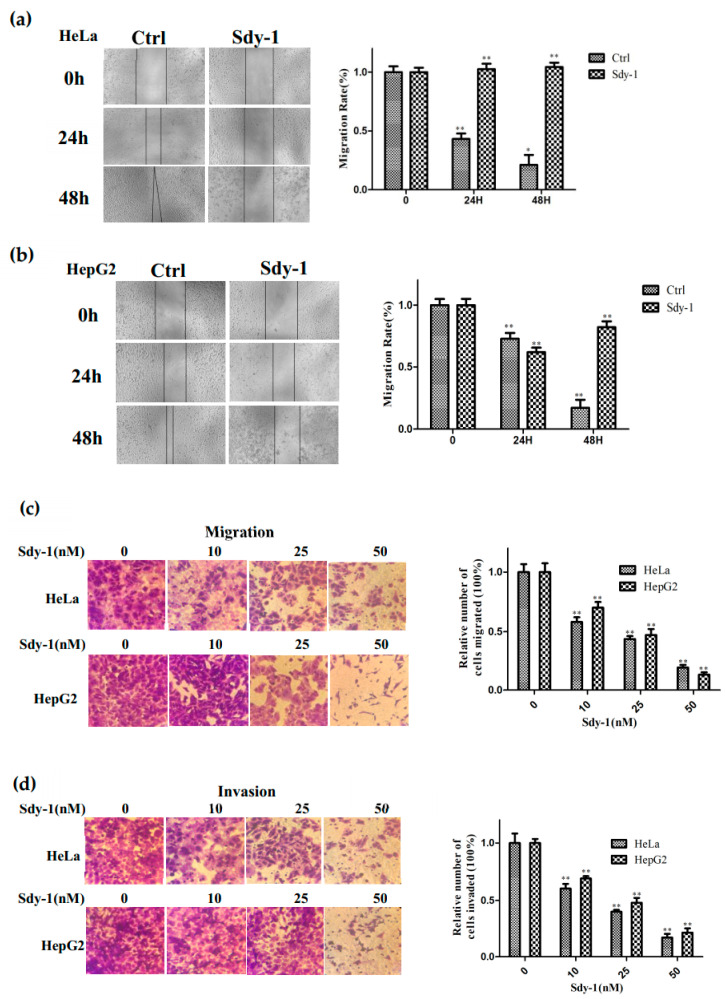
Sdy-1 suppresses migration and invasion of HeLa and HepG2 cells. Sdy-1 inhibits migration of HeLa (**a**) and HepG2 (**b**) cells. The cell monolayer was scraped off and incubated with 10 nM Sdy-1 for 24 h and 48 h before photomicrographs. After treatment for 24 h, the cells were subjected to transwell assays (200×). Migration rate (**c**) and invasion rate (**d**) were measured (n = 3). * *p* < 0.05, ** *p* < 0.01 vs. the control group.

We tested the effect of Sdy-1 on the migration of the two cell types by the cell scratch test and transwell assays. As shown in Figure 2a,b, the size of the wound area after treatment is significantly larger than that of the untreated group, and the healing rate is slower as the drug concentration increases. Sdy-1 inhibited the migration of HepG2 and HeLa cells in a dose-dependent manner (Figure 2c). To further examine the effect of Sdy-1 on HepG2 and HeLa cells invasion, we performed transwell assays using Matrigel coated chambers. Compared with the blank group, the number of penetrated HepG2 and HeLa cells in the treatment groups with different concentrations of Sdy-1 decreased in a gradient compared with the blank control group (Figure 2d). These results indicated that Sdy-1 inhibited the migration and invasion abilities of HepG2 and HeLa cells in vitro. 

### 2.3. Sdy-1 Induces Cellular Apoptosis

**Figure 3:**
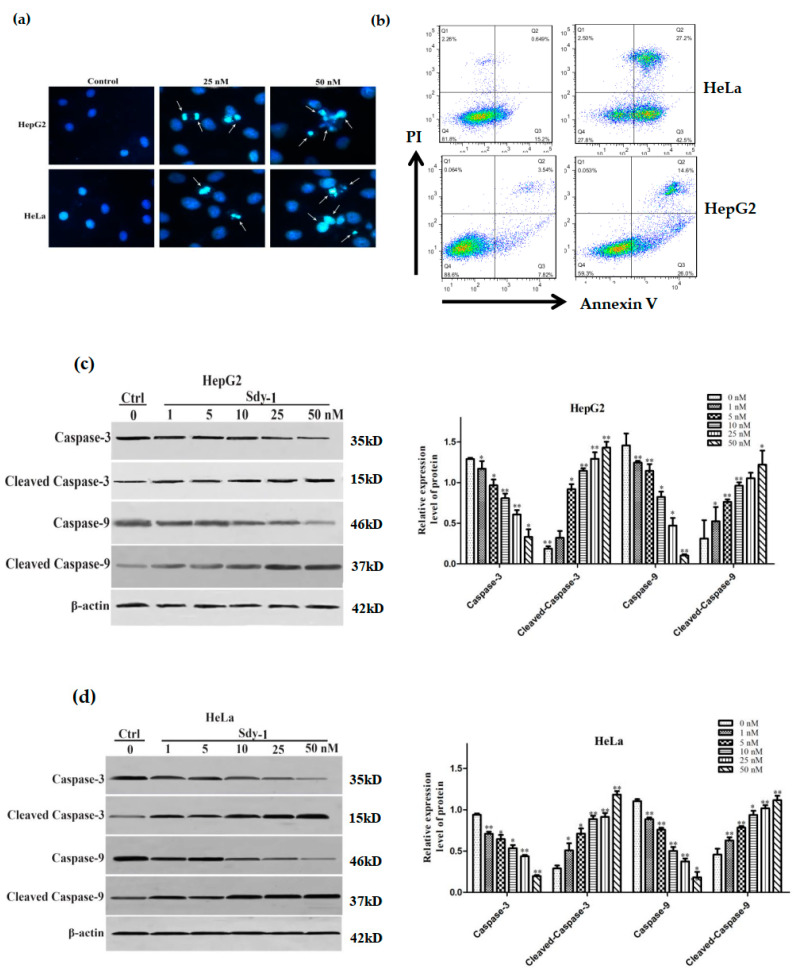
Sdy-1 induces cellular apoptosis. (**a**) HepG2 and HeLa were treated with two concentrations of Sdy-1 (25 or 50 nM) or 0.1% DMSO for 24 h, and apoptosis was observed by Hoechst 33258 staining. (**b**) Cell apoptotic death analyzed by Annexin V/PI staining. Cells were treated with 10 nM of Sdy-1 for 48h, stained with Annexin V-FITC and PI for 30 min and analyzed by flow cytometry. Annexin V positive populations are considered as cells undergoing apoptosis. HepG2 (**c**) and HeLa (**d**) cells were treated with different concentrations of Sdy-1 (1, 5, 10, 25, or 50 nM) or 0.1% DMSO for 24 h. The expression of Caspase-3, Caspase-9, cleaved Caspase-3 and cleaved Caspase-9 were observed using Western blotting. Data are expressed as a mean ± SD of three independent experiments. * *p* < 0.05, ** *p* < 0.01 vs. the control group.

The apoptosis-inducing effect of Sdy-1 was investigated by nuclear staining with Hoechst 33258. As shown in Figure 3a, chromatin condensation, nuclear fragmentation, and apoptotic bodies were clearly observed in the cells after treatment. In Figure 3b after flow cytometry assay, it was found that HeLa and HepG2 cells were treated with Sdy-1 for 48 h, the number of Annexin V-positive cells increased from 15.20%, 7.82% to 42.50% and 26.00%, and Annexin V-positive cells increased significantly, suggesting that Sdy-1 promoted tumor cell apoptosis. We also performed detection of the Caspase pathway in HepG2 and HeLa cells by Western blot. As shown in Figure 3c,d, Sdy-1 downregulated Caspase-3 and -9 proteins and upregulated cleaved Caspase-3 and cleaved Caspase-9 proteins.

### 2.4. Sdy-1 Induces G1 Arrest in HepG2 Cells

**Figure 4:**
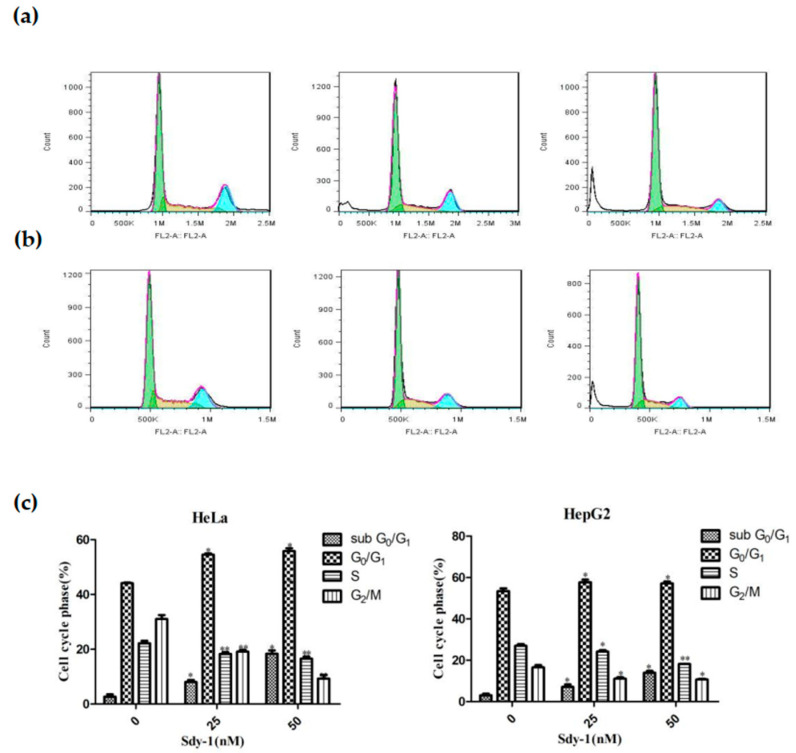
Flow cytometry detection of the effects of Sdy-1 on tumor cell cycle arrest. HeLa (**a**) and HepG2 (**b**) cells were stimulated with 25 and 50 nM Sdy-1 for 48 h, and then stained with PI for 30 min at room temperature. Phases of the cell cycle were measured by flow cytometry. (**c**) A statistical investigation was performed for changes of HeLa and HepG2 cell cycle at 48 h after Sdy-1 treatment. Data are expressed as a mean ± SD of three independent tests. * *p* < 0.05, ** *p* < 0.01 vs. the control group.

Our previous study showed that Sdy-1 induced cell-cycle arrest at G_0_/G_1_ phase in a time-dependent increment in tumor cells [28]. Herein, we used flow cytometry to detect cell cycle distribution of HeLa and HepG cell lines with the treatment of different concentrations of Sdy-1 (0–50 nM without FBS) after 48 h. We found that Sdy-1 induced accumulation of the G_0_/G_1_ phase in tested tumor cells accompanied by a decrease in S and G_2_/M phases was in line with a dose-dependent manner of Sdy-1. The percentage of tested tumor cells arrested in G_0_/G_1_ phase with treatment of Sdy-1 were 44.14 ± 0.23%, 54.55 ± 0.42% and 55.81 ± 1.04% (in HeLa cells, Figure 4a) and 53.34 ± 1.31%, 57.73 ± 1.24% and 57.17 ± 0.88% (in HeLa cells, Figure 4b) at 0, 25, and 50 nM concentration of Sdy-1, respectively. 

The sub-G_0_/G_1_ peak eventually shifted to a hypodiploid sub-G_0_/G_1_ peak to 18.35 ± 1.21% of HeLa cells and 13.98 ± 0.86% of HepG cells after 48 h Sdy-1 treatment at 50 nM concentration, respectively, compared to untreated cells at the same phase (2.65 ± 0.88% of HeLa cells and 3.05 ± 0.78% of HepG2 cells) (Figure 4c). 

### 2.5. Sdy-1 Changes the Level of β-Catenin in the Cells

**Figure 5:**
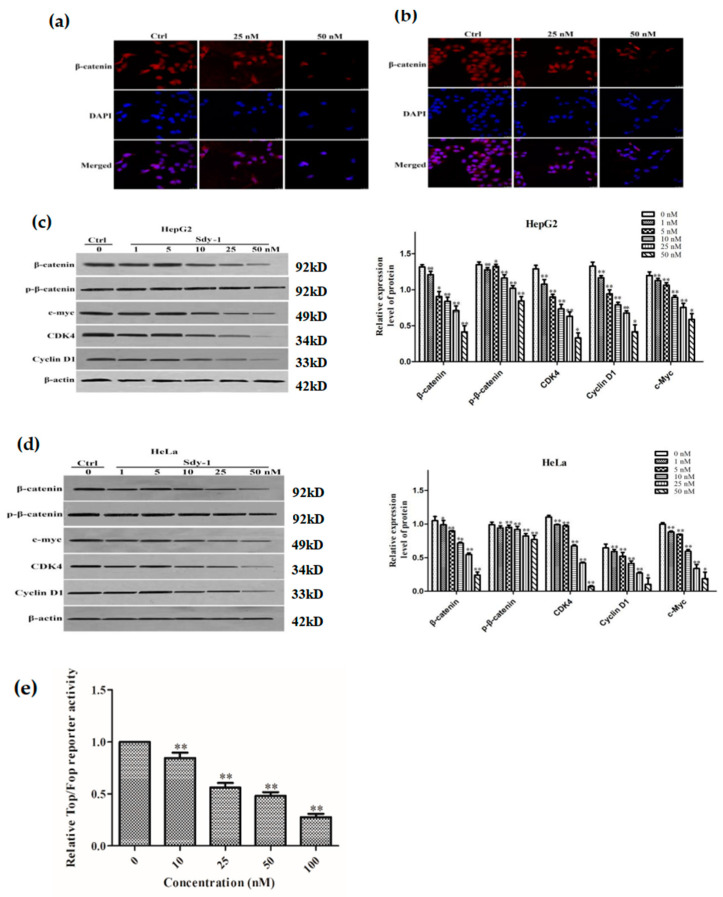
Sdy-1 alters the level of β-catenin in cells on Wnt signaling pathway. After treatment of HepG2 (**a**) and HeLa (**b**) cells with 25 nM and 50 nM Sdy-1 for 24h, the levels of β-catenin were changed in the cells. β-catenin was determined by immunocytochemical staining using Cy3 goat anti-rabbit IgG. Whole HepG2 (**c**) or HeLa (**d**) cell lysates were prepared and the levels of β-catenin, p-β-catenin, CDK4, c-myc, and Cyclin D1 were determined by Western blot analysis and normalized to β-actin. (**e**) HEK-293T cells were seeded into 24-well plate at a density of 5 × 104 cells/well, and Top Flash or Fop Flash and pRL-TK were transfected into the cells using ExFect2000 transfection reagent. After transfection for 24 h, different concentrations of Sdy-1 or 0.1% DMSO were treated for 24 h and then tested using the Dual Luciferase Reporter Assay Kit. Representative data from three independent experiments are shown. * *p* < 0.05, ** *p* < 0.01 vs. the control group.

We investigated the localization of β-catenin, a key protein in Wnt signaling in cells, y immunofluorescence. The results are shown in Figure 5a,b. The content of β-catenin in HepG2 and HeLa cells diminished significantly with increasing Sdy-1 concentration in cells. The results indicate that Sdy-1 has a tendency to reduce the distribution of β-catenin. This was a preliminary hint that Sdy-1 inhibition of tumor cell proliferation may be related to its hindrance of the Wnt signaling pathway.

### 2.6. Sdy-1 Inhibits the Wnt Signaling Pathway

To further demonstrate the inhibitory impact of Sdy-1 on the Wnt pathway, we used TOPFlash or FOPFlash to transfect in HEK-293T cells and Western blotting to detect the levels of β-catenin, p-β-catenin and its downstream proteins CDK4, cyclin D1, and c-myc in HepG2 and HeLa cells. The expression of downstream related proteins also decreased accordingly, indicating that Sdy-1 acts on the Wnt signaling pathway, it does affect the downstream of the Wnt signaling pathway (Figure 5c,d). Meanwhile, as the drug concentration increased, the fluorescence value of the cells decreased significantly, indicating that Sdy-1 has a significant inhibitory effect on the Wnt signaling pathway and the Western blot result displayed that the expression of total β-catenin protein was significantly reduced, and the level of p-β-catenin did not increase. The results suggest that, but it may not affect the upstream of the Wnt signaling pathway and may not affect the Wnt signaling pathway through the cascade reaction that affects the Wnt signaling pathway from top to bottom (Figure 5c–e). 

### 2.7. Sdy-1 Inhibits Transcription of the β-Catenin Gene in Cells

**Figure 6:**
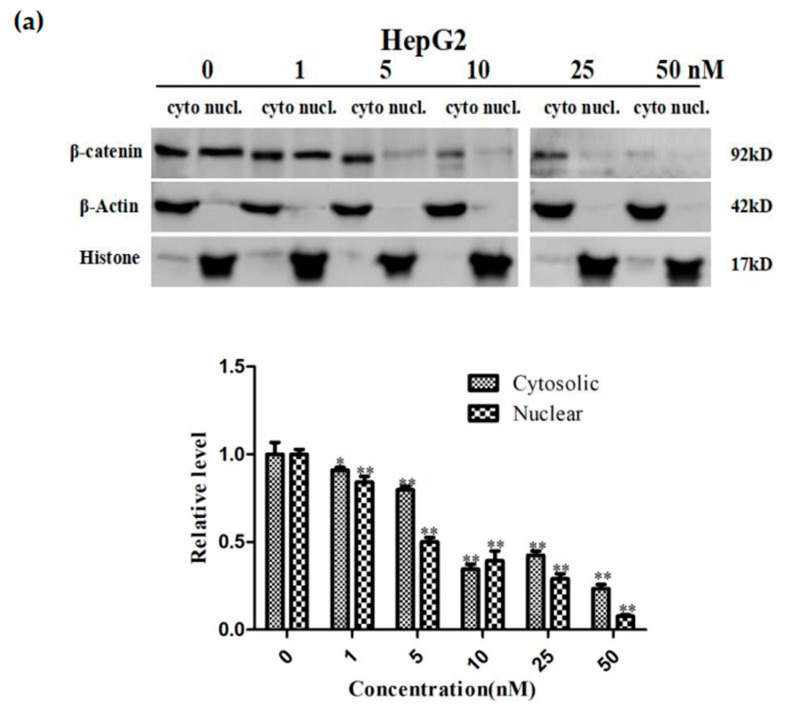

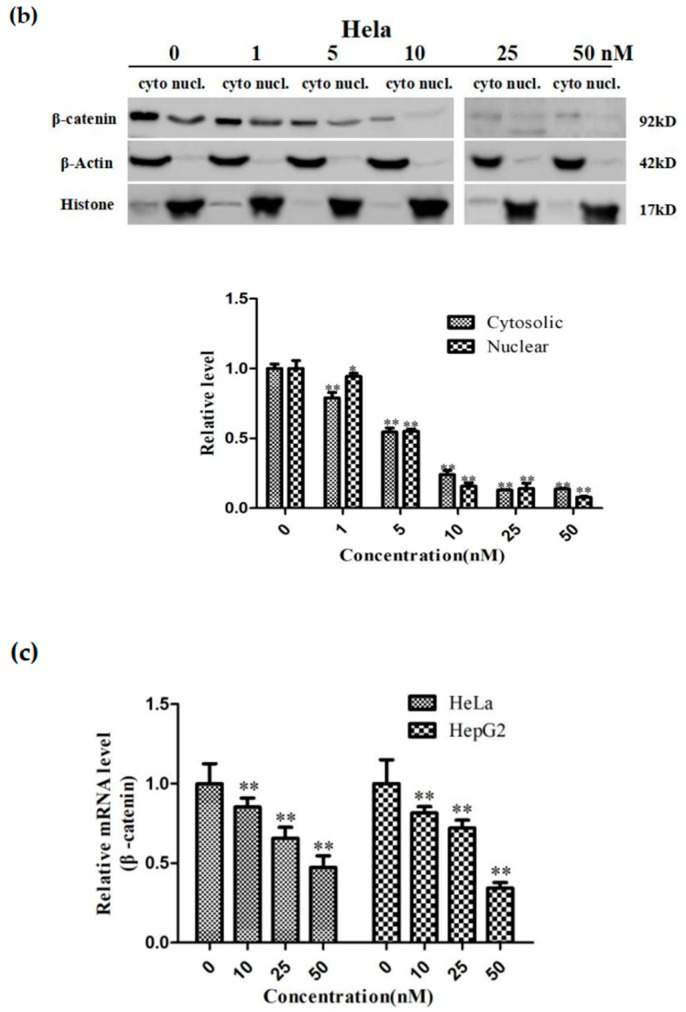
Effect of Sdy-1 on β-catenin gene synthesis in HepG2 and HeLa cells. (**a**) HepG2 and (**b**) HeLa cells were treated with 10, 25, or 50 nM Sdy-1 for 24 h. Cytoplasmic and nuclear lysates from HepG2 and HeLa cells, β-catenin levels were determined by Western blot analysis. (**c**) The mRNA expression of β-catenin after Sdy-1 treatment was detected by real-time quantitative PCR. Sdy-1 markedly reduced transcription of β-catenin in a dose-dependent manner. Data are expressed as mean ± SD of three independent tests. * *p* < 0.05, ** *p* < 0.01 vs. the control group.

We investigated β-catenin levels in lysates of both tumor cell cytosol and nucleus after 24 h of Sdy-1 treatment (Figure 6a,b). The results showed that Sdy-1 inhibited the level of β-catenin in the cytoplasm and nuclear lysate of two kinds of tumor cells. Meanwhile, the treatment of HepG2 and HeLa cells with Sdy-1 clearly decreased the accumulation of β-catenin in the nucleus, which further confirmed the inhibition of Sdy-1 on nuclear translocation of β-catenin. As shown in Figure 6c, q-PCR showed that the expression level of the β-catenin gene decreased with increasing concentration. The effect was obvious at 50 nM. The decrease of mRNA means that the transcription of the β-catenin gene is reduced. This suggested that Sdy-1 may inhibit Wnt signaling by directly interfering with β-catenin mRNA transcription in the nucleus.

### 2.8. Molecular Docking Analysis of the Binding Interaction of Sdy-1 with β-Catenin

**Figure 7:**
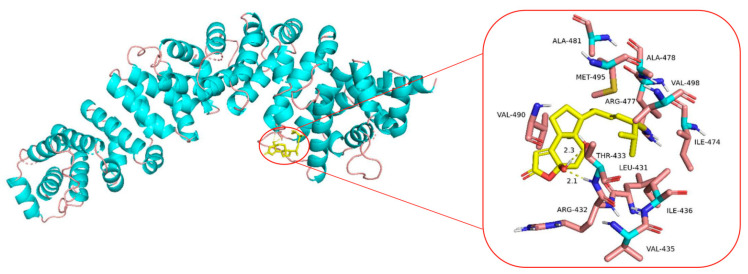
Molecular docking analysis of Sdy-1 binding to β-catenin. Molecular docking between Sdy-1 and β-catenin receptor was simulated using the AutoDock 4.2 program. The hydrogen bonds are indicated by yellow dashed lines.

Molecular docking was then performed to further understand the possible binding modes and binding affinities of the highly active Sdy-1 with the active sites of the β-catenin using Autodock 4.2. As shown in Figure 7, Sdy-1 formed two key hydrogen bonds with residue THR-433 with the binding energy of −5.92 kcal·mol^−1^. Meanwhile, Sdy-1 formed hydrophobic interaction with residue ILE-436, ILE-474, ARG-477, VAL-490, VAL-498 and intermolecular interaction with residue LEU-431, ARG-432, PRO-434, ALA478, ALA-481, MET-495.

### 2.9. Sdy-1 Inhibits Xenograft Tumor Model

**Figure 8:**
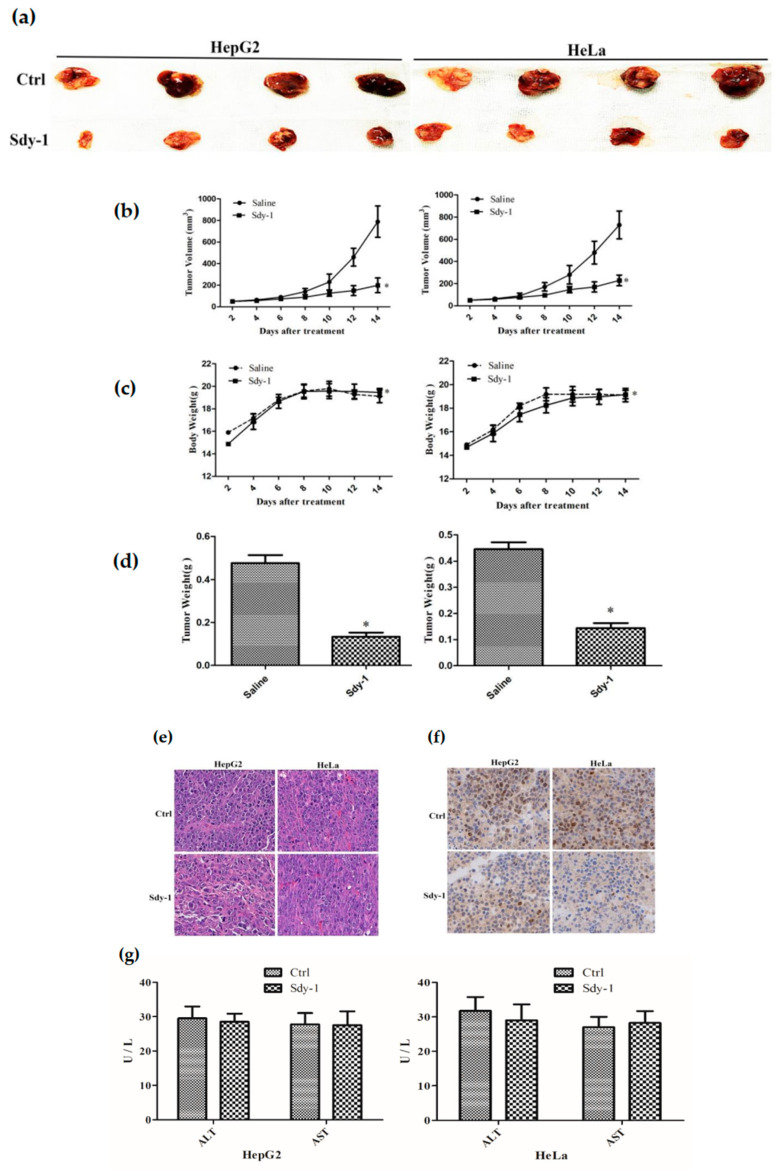
Effect of Sdy-1 on the xenograft model in nude mice. HepG2 and HeLa were injected into nude mice and then treated with drugs. On the next day, Sdy-1 (n = 4) or saline (n = 4) was intravenously injected at a dose of 0.1 mg/kg every other day for consecutive 14 days. The nude mice were sacrificed by cervical dissection to observe the tumor morphology (**a**). Mean tumor volume (**b**). Mean body weight (**c**). Mean tumor weight (**d**). And performed HE staining (**e**), immunohistochemical experiments to detect changes in Ki67 (**f**) and ALT/AST analysis (**g**). Representative data from six independent experiments are shown. * *p* < 0.05 vs. the control group.

To validate the role of Sdy-1 in vivo, we constructed a nude mouse xenograft model. As shown in Figure 8a–d, after two weeks of treatment, the tumor volume of the treatment group is significantly smaller than that of the control group. HE staining showed that Sdy-1 significantly reduced the density of tumor cells (Figure 8e). In addition, Sdy-1 was found to significantly reduce the level of the cell proliferation marker Ki67 by immunohistochemical analysis (Figure 8f). These results indicate that Sdy-1 has an inhibitory effect on tumor cell growth. Moreover, there was no change in the morphology of hepatocytes in the Sdy-1 group, and the corresponding ALT/AST expression did not change (Figure 8g). 

## 3. Discussion

**Figure 9:**
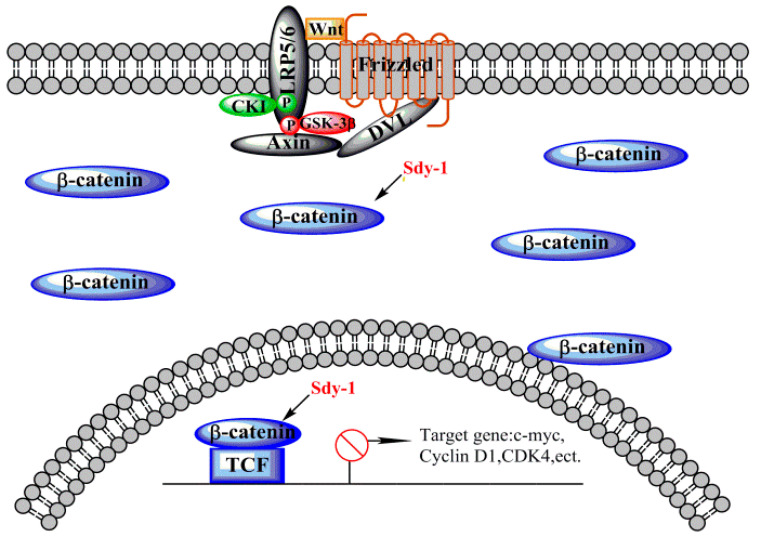
Sdy-1 acts on the Wnt signaling pathway. When the Wnt pathway is activated, β-catenin is in a free state and enters the nucleus to bind to TCF and activate downstream target genes. Sdy-1 can reduce free β-catenin and prevent transcription of downstream target genes.

The mechanism by which the Wnt signaling pathway is involved in tumorigenesis involves cell proliferation and migration, cell cycle, and apoptosis [33]. Therefore, the development of anti-tumor drugs based on the Wnt signaling pathway is of great significance. Studies have shown that Sdy-1 can effectively inhibit the Wnt signaling pathway at nanomolar concentrations. As shown in Figure 9, Sdy-1 may inhibit the Wnt pathway by directly inhibiting β-catenin gene transcription instead of the cascade reaction from top to bottom in HepG2 and HeLa. At the same concentration level, Sdy-1 can inhibit the growth, migration and invasion of HepG2 and HeLa, it promotes apoptosis and causes G1 phase arrest in the HeLa cells cycle. In addition, in nude mouse xenograft models, Sdy-1 also shows good antitumor activity in vivo.

In previous reports, Sdy-1 acts as a selective protein inhibitor that inhibits human protein tyrosine phosphatase SHP2-dependent cellular signaling [34,35]. It also affects the migration of tumor cells by activating the tyrosine-protein kinase SRC family and downstream targets [36]. Sdy-1 has been used as a potential immunosuppressive agent to inhibit tumor necrosis factor-α, interleukin-2 (IL-2), and IL-4 mRNA expression in human peripheral blood mononuclear cells (PBMCs) activated by phytohemagglutinin (PHA) while reducing the expression of cell proliferation factors NF-AT and NF-κB, as well as upstream cell calcium ion concentration and protein kinase C activity [30]. Flow cytometry assay suggests that sdy-1 promoted tumor cell apoptosis, and speculate that it may promote apoptosis by downregulating Caspases-3 and -9. We have also proven that it can inhibit the cell cycle [28]. Here, we show that Sdy-1 exerts antitumor activity by inhibiting the Wnt signaling pathway.

When the Wnt pathway is not activated, β-catenin forms a protein complex with APC, Axin, GSK-3β, etc. [37], GSK-3β phosphorylates β-catenin, thereby promoting the protease ubiquitination to degrade phosphorylated β-catenin [38,39]. Moreover, another part of β-catenin is adhered to by E-cadherin so that β-catenin has always been at a lower level in the cytoplasm [40]. Therefore, in cells where the Wnt pathway is not activated, p-β-catenin often has high levels in the cytoplasm and if the Wnt signaling pathway is inhibited by the upstream Axin/GSK-3β/APC or Frizzled associated proteins, p-β-catenin will also be at a higher level in the cytoplasm [41]. We found that Sdy-1 can reduce the expression of the total β-catenin protein in the cell and did not increase the expression of p-β-catenin, but decreased slightly, that is, Sdy-1 may have no effect on the cascade reaction prior to the phosphorylation of β-catenin, so we speculate that Sdy-1 has little effect on the upstream of the Wnt signaling pathway.

Then we consider the effect of Sdy-1 on the downstream of the Wnt signaling pathway. The free β-catenin protein in the cytoplasm accumulates in the cytoplasm, finally enters the nucleus, and combines with the transcription factor TCF/LEF to activate the transcriptional activities of target genes such as c-myc, Cyclin D1, CDK4, etc., thereby affecting the cell’s proliferation and differentiation lead to canceration [42,43]. Therefore, we used real-time quantitative PCR technology to detect β-catenin genes in HepG2 and HeLa cells after Sdy-1 administration. It was found that compared with the control group, Sdy-1 reduced β-catenin gene transcription in both tumor cells, which indicates that Sdy-1 can inhibit the β-catenin gene transcription level, this results in a decrease in the synthesis of the β-catenin protein during the translation of β-catenin. This also echoes the previous experimental results that Sdy-1 reduced the total intracellular β-catenin protein expression and explained why the p-β-catenin protein expression did not increase but decreased. Therefore, we believe that Sdy-1’s inhibition of the Wnt signaling pathway is related to Sdy-1’s inhibition of β-catenin gene synthesis. Our team also plans to detect the upstream proteins of the Wnt signaling pathway in future studies to further prove this conclusion.

Molecular docking has also been used to explore the behavior of small molecules in the binding site of a target protein on the basis of the shape, binding affinity and drug score [44]. There are some experimental known inhibitors that were docked against Wnt signaling-related targets which were consistent with the experimental data, but very few of them were targeted β-catenin directly [45,46]. In this study, AutoDock 4.2 was adopted to perform molecular docking to propose the hypothetic mechanism underlying the inhibitory activity of Sdy-1 against β-catenin. The docking results suggested that Sdy-1 could exhibit inhibitory effects towards β-catenin through binding to an active site on THR-433, which deserved further investigation. 

## 4. Materials and Methods

### 4.1. Reagents

Sdy-1 was isolated from the *R. mucronata* endophytic *Pestalotiopsis* sp. HQD-6, which is a typical mangrove plant as described previously [28]. 3-(4,5-dimethylthiazol-2-yl)-2,5-diphenyltetrazolium bromide (MTT) was purchased from Sigma-Aldrich, St. Louis, Missouri, USA. Giemsa dye solution was obtained from Biosharp (Wuhan, China). Minimum Eagle’s Medium (MEM) was obtained from Real Times Biotechnology Co. Ltd. (Beijing, China). Total RNA Extraction Reagent, HiScript 1st Strand cDNA Synthesis Kit, 2 × tap Master Mix and Annexin V-FITC/PI Apoptosis Detection Kit were purchased from Vazyme (NanJing, China). Antibodies against Caspase-3, cleaved Caspase-3, Caspase-9, cleaved Caspase-9, β-catenin, p-β-catenin, c-myc, CDK4, Cyclin D1, β-actin, HRP goat anti-mouse IgG and Cy3 goat anti-rabbit IgG were purchased from Boster Biological Technology Co. Ltd. (Wuhan, China). Hoechst33258 staining kit was purchased from Biyuntian Biotechnology Co., Ltd. (Shanghai, China). Propidium Iodide (PI) was from Biofroxx (Duisburg, Germany). The reporter plasmids Top Flash, Fop Flash, pRL-TK were from Miaoling Biological Technology Co., Ltd. (Wuhan, China). Other chemical reagents were purchased from Guangzhou chemical reagent factory (Guangzhou, China).

### 4.2. Cell Culture

HepG2 (liver cancer cell line), HeLa (cervical cancer cell line) and HEK-293T (human kidney cell line) were purchased from the Type Culture Collection of the Chinese Academy of Sciences, Shanghai, China. The rat liver BRL-3A cells were purchased from ATCC. HepG2, HeLa and HEK-293T were cultured in MEM, and BRL-3A was cultured in DMEM containing 10% FBS, 1% penicillin-streptomycin, then these cells were cultured in the presence of 5% CO_2_ at 37 °C. All cells were used for fewer than 6 months after resuscitation.

### 4.3. MTT Assay

Cell viability was detected by the MTT method. BRL-3A cells in the logarithmic growth phase were digested with trypsin and plated in 96-well plates at a cell density of 5000 per well and underwent 24 h Sdy-1 treatments, then incubated in 0.5 mg/mL MTT for 4 h at 37 ℃. Upon removal of the medium, 200 μL DMSO was added to dissolve the MTT formazan crystals. After shaking gently on the shaker, the absorbance of the plate was then read at 570 nM using a Microplate reader.

### 4.4. Colony Formation Assay

For colony formation assay, cells (2 × 10^2^ cells/well) were trypsinized and plated in 6-well plates in MEM medium with 10% fetal bovine serum incubated at 37 ℃ in 5% CO_2_ for 24 h. Then adherent cells were dealt with different concentrations of Sdy-1 (5, 10, 50, 100, and 500 nM) or 0.1% DMSO. After the plates were incubated at 37 ℃ in 5% CO_2_ for 14 days, the colonies were fixed and stained with Giemsa dye solution stain for 30 min. Finally, cells were washed with sterile water and dried under air. The number of colonies of more than 50 cells was counted and photographed. 

### 4.5. Scratch-Wound Assay

HepG2 and HeLa cells (2 × 10^5^ cells/well) were seeded in a six-well plate and grown overnight. Scrape the cell monolayer in a straight line to create a “scratch” with a pipet tip and the scratched cells were removed by washing with PBS. Sdy-1 (10 nM) or 0.1% DMSO was added and migration of the cell monolayer from the edge of the scratch was recorded by measuring the distance at 0, 24 and 48 h under a microscope.

### 4.6. Transwell Assays

Cells (2 × 10^5^ cells/well) were seeded in 24 transwell chambers with 8-μm pore membrane in a serum-free medium in the absence and presence of Sdy-1 (10, 25, 50 nM). Medium with 20% FBS was added to the bottom chamber. Cells were incubated in a 5% CO_2_ and 37 °C incubator for 6 h and then fixed with 4% PFA. The upper luminal cells were removed with cotton swabs, stained with 0.1% crystal violet, and then photographed microscopically. Bound crystal violet was dissolved with 33% acetic acid and absorbance was measured at 570 nm for quantitative analysis. For invasion assays, the chambers with 8-μm pore membranes were coated with 20 μL Matrigel. Other procedures were the same with the migration assay.

### 4.7. Analysis of Cell Apoptosis

Hoechst 33258 staining was used to quantitatively determine the number of cells undergoing apoptosis. Cells (2 × 10^5^ cells/well) were seeded in 6-well plates and incubated overnight to allow attachment. The cells were treated with Sdy-1 at different concentrations (25, 50 nM) or 0.1% DMSO for HepG2 and HeLa cells for 24 h and then fixed with 4% PFA for 10 min. Cells were then stained with Hoechst 33258 (0.5 mL) for 5 min and washed twice with PBS. A drop of fluorescence quencher was added before being subjected to a fluorescence microscope.

Annexin V-FITC/PI dual staining analysis is used for the detection of the apoptosis rate. Cells were plated in 6-well culture plates (5 × 10^4^ cells/well) and underwent 48 h Sdy-1 treatments, after which the cells were washed with PBS and trypsinized. Then, the staining dye of Annexin V (5 μL) and PI (5 μL) was added to cells and incubated in the dark at 4 °C for 30 min. At last, the stained cells were analyzed using a flow cytometer.

### 4.8. Cell Cycle Analysis

The cell cycle was assessed by flow cytometry using the propidium iodide (PI) staining. Cells were seeded at a density of 2 × 10^5^ cells/well in 6-well culture plates in the presence of 25, 50 nM Sdy-1 or 0.1% DMSO. After incubation at 37 °C for 48 h, the adherent cells were washed once with PBS, harvested with trypsin, collected by centrifugation (1,000 rpm for 5 min), and washed twice with cold PBS. Then, the cells were stained with 400 μL PI (50 μg/ mL) for 30 min at room temperature in the dark. After incubation, the cell-cycle kinetics were analyzed by flow cytometry at 488 nm excitation using FlowJo software.

### 4.9. Luciferase Reporter Gene Assay

Luciferase assays were performed with the Luciferase assay systems kit Dual Luciferase Reporter Assay Kit (vazyme) according to the manufacturer’s instructions. In brief, HEK-293T cells were seeded in 24-well plates at a density of 5 × 10^4^ cells/well and transfected with TopFlash or FopFlash (0.5 μg) and internal control plasmid pRL-TK (0.5 μg) using ExFect2000 (vazyme). After transfection for 24 h, Sdy-1 (10, 25, 50, 100 nM) or 0.1% DMSO were added and the cells incubated for 24 h, lysed in 1× passive lysis buffer (vazyme), and collected for luciferase activity assay. Firefly luciferase activity was normalized for transfection efficiency by Renilla luciferase activity.

### 4.10. Immunocytochemical Staining

Cells at a density of 2 × 10^4^ cells/well were seeded in 6-well plates and dealt with different concentrations of Sdy-1 (25, 50 nM) or 0.1% DMSO for 24 h. After treatment, cells were fixed with 4% paraformaldehyde for 30 min, followed by permeabilization in 0.5% Triton X-100 for 15 min and blocked for 1 h with 5% BSA, and then incubated with anti-β-catenin primary antibodies overnight. The corresponding fluorescent secondary antibody was hatched for 1 h at room temperature within the dim. Nuclear staining was performed with the addition of a drop of DAPI overnight. Stained cells were washed with PBS and visualized using confocal microscopy.

### 4.11. Western Blot Analysis

After dealing with Sdy-1 (1, 5, 10, 25, 50 nM) or 0.1% DMSO for 24 h, cells were collected and lysed in a RIPA buffer containing PMSF. The protein concentration was subjected to BSA Protein Assay Kit. The nuclear and cytoplasmic proteins were extracted using the cell structure nuclear and cytoplasmic protein extraction kit according to the manufacturer’s protocol. The extracted proteins were quantitated and denatured with 5× SDS-PAGE sample loading buffer by boiling for 10 min, subsequently separated on 10% sodium dodecyl sulfate polyacrylamide gel electrophoresis (SDS-PAGE) gels, and then transferred onto polyvinylidene fluoride (PVDF) membranes and blocked with 5% skimmed milk powder for 1 h. The primary antibodies against Caspase 3, cleaved Caspase 3, Caspase 9, cleaved Caspase 9, Cyclin D1, CDK4, β-catenin, p-β-catenin, c-myc or β-actin was diluted with TBST containing 5% skim milk powder and the PVDF membrane was overlaid on the diluted antibody for 2 h at normal temperature. After the membranes were then washed with TBST three times and incubated with the diluted secondary antibody for 1 h at normal temperature. The luminescent liquid was added and incubated for 3 min, the membranes were placed in plastic sleeves and exposed to film in the dark.

### 4.12. q-PCR Analysis

HepG2 and HeLa cells (4 × 10^5^ cells/dish in 60-mm dishes) for 24 h, and then cells were treated with different concentrations of Sdy-1 (10, 25, 50 nM) or 0.1% DMSO for 12 h. Total RNA was extracted with TRIzol reagent and reverse transcribed using the HiScript First Strand cDNA Synthesis Kit with random primers according to the manufacturer’s instructions. Q-PCR was performed with SYBR Green qPCR Master Mix (High ROX) using ABI StepOne Plus Real-time Detection System and Glyceraldehyde-3-phosphate dehydrogenase (GAPDH) was used as the internal control. Primers were:

Human β-catenin forward primer: 5′-TGCCAAGTGGGTGGTATAGAGG-3′ and the reverse primer: 5′-CGCTGGGTATCCTGATGTGC-3′.

Human GAPDH forward primer: 5′-CCAGAACATCATCCCTGCCTCTACT-3′ and the reverse primer: 5′-GGTTTTTCTAGACGGCAGGTCAGGT -3′.

### 4.13. Molecular Docking Analyses

Molecular docking studies were performed to investigate the binding mode of Sdy-1 with β-catenin using AutoDock 4.2 software. The crystallographic structure of β-catenin (PDB-ID: 4r0z) was obtained from Research Collaboratory for Structural Bioinformatics Protein Data Bank (RCSB PDB, http://www.rcsb.org, 21 June 2021). The β-catenin protein was protonated and deleted water at pH 7 using the Clean Protein tool. The 3D structure of the small molecule was built and optimized by using Chem3D Ultra 14.0 software. The steepest gradient algorithm using the MM2 force field was applied for energy minimization to generate the best conformation. A docking site was defined as all residues within RMS tolerance of 1.0 Å. All other parameters were set as default. The binding energies between β-catenin and Sdy-1 were calculated by AutoDock 4.2 software with the Lamarckian genetic algorithm method [47]. The visual analysis of the best-scoring pose was shown using PyMOL software (Schrödinger, LLC: NY, USA) [48]. 

### 4.14. Nude Mouse Xenograft Model

All animal experiments were performed according to the protocols approved by the ethical review board of the Administrative Committee on Animal Research of Hainan University (Haikou, China), and the project identification code is HNUAUCC-2021-00082. BALB/c nude mice (4–5 weeks old) were purchased from Changsha Tianqin Biotechnology Co., Ltd. (Changsha, China) HepG2 or HeLa cells were injected into the right forelimb of nude mice at a density of 5 × 10^7^ cells/mL (150 µL). Once the tumor volume reached about 50 mm^3^, the mice were randomly divided into two groups with the treated group (n=4) injected with Sdy-1 (0.1 mg/kg/2day) and a control group (n = 4) injected with an equal volume of saline. Subsequently, tumor volumes are measured with a caliper and calculated according to the formula: length × width^2^/2. After treatment for two weeks, the mice were sacrificed by cervical dislocation and tumor samples were retrieved for histological evaluation. Then, the tumor tissue was stripped and formalin-fixed, paraffin-embedded, cut into 4-μm sections, and immunohistochemically stained with hematoxylin and eosin (H&E). Anti-Ki-67 (Cell Signaling Technology Co., Ltd.: Danvers, MA, USA) immunohistochemical analysis and ALT, AST detection were performed.

### 4.15. Statistical Analysis

Statistical analysis of the data was performed by one-way ANOVA followed by Dunnett’s t test (SPSS 13.0 software, SPSS, New York, NY, USA), the difference was considered significant at the *p* < 0.05 level, and the data were expressed as mean ± standard deviation.

## 5. Conclusions

In summary, this study found that mangrove endophytic fungi originated Sdy-1 is a novel inhibitor of the Wnt signaling pathway. Sdy-1 effectively inhibits the abnormal activation of the Wnt signaling pathway by inhibiting the gene transcription of β-catenin. This study revealed the new mechanism of the anti-tumor effect of Sdy-1, providing a theoretical basis for the treatment of liver cancer and cervical cancer.

## Supplementary Materials

The following supporting information can be downloaded at: https://www.mdpi.com/article/10.3390/md20020125/s1, Figures S1–S8: NMR spectroscopic experiments results and mass spectrometry data. Figure S1: ^1^H-NMR of Sdy-1, Figure S2: ^13^C-NMR of Sdy-1, Figure S3: DEPT of Sdy-1, Figure S4: ^1^H-^1^H COSY of Sdy-1, Figure S5: HMQC of Sdy-1, Figure S6: HMBC of Sdy-1, Figure S7: NOESY of Sdy-1, Figure S8: ESI-MS of Sdy-1.
