# Supplementary material for: Sdy-1 Executes Antitumor Activity in HepG2 and HeLa Cancer Cells by Inhibiting the Wnt/β-Catenin Signaling Pathway
Source: Mar Drugs. 2022 Feb 5;20(2):125. doi: 10.3390/md20020125 (PMC8877534; doi:10.3390/md20020125)
Supplement: Supplementary file 1 [file marinedrugs-20-00125-s001.zip › marinedrugs-1496727-supplementary.pdf]

Sdy-1 executes antitumor activity in HepG2 and Hela cancer cells by inhibiting the Wnt/β-catenin signaling pathway

Mengyu Sun [1,‡], Dongdong Zhou [1,‡], Jingwan Wu[1], Jing Zhou [2] and Jing Xu [1,*]

[1] One Health Institute, School of Chemical Engineering and Technology, Hainan University, Haikou 570228, China; summer@hainanu.edu.cn (M. S.); dongdongchoy@hainanu.edu.cn (D. Z.); 2021110817000019@hainanu.edu.cn (J. W.)

[2] School of Life Sciences, Hainan University, Haikou 570228, China; 993725@hainanu.edu.cn (J. Z.)

* Correspondence: happyjing3@hainanu.edu.cn (J. X.)

‡ These authors contributed equally to this work.

Contents

Figure S1. ^{1}H-NMR of Sdy-1 3

Figure S2. ^{13}C-NMR of Sdy-1 3

Figure S3. DEPT of Sdy-1 4

Figure S4. ^{1}H-^{1}H COSY of Sdy-1 4

Figure S5. HMQC of Sdy-1 5

Figure S6. HMBC of Sdy-1 5

Figure S7. NOESY of Sdy-1 6

Figure S8. ESI-MS of Sdy-1 6

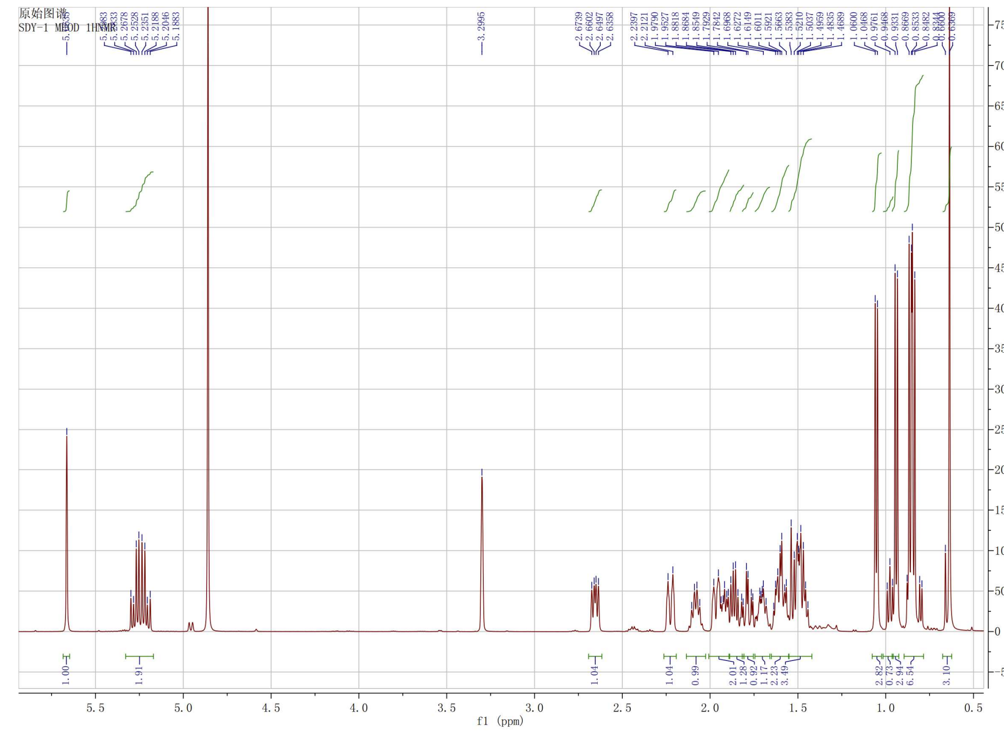

Figure S1. ^{1}H-NMR of Sdy-1

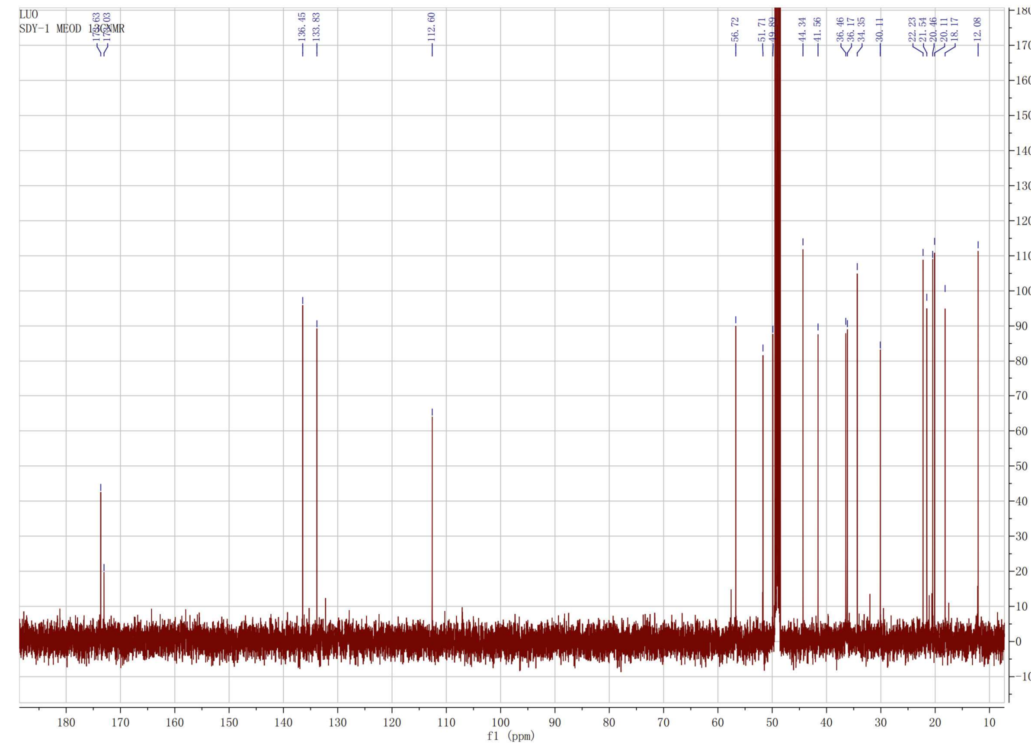

Figure S2. ^{13}C-NMR of Sdy-1

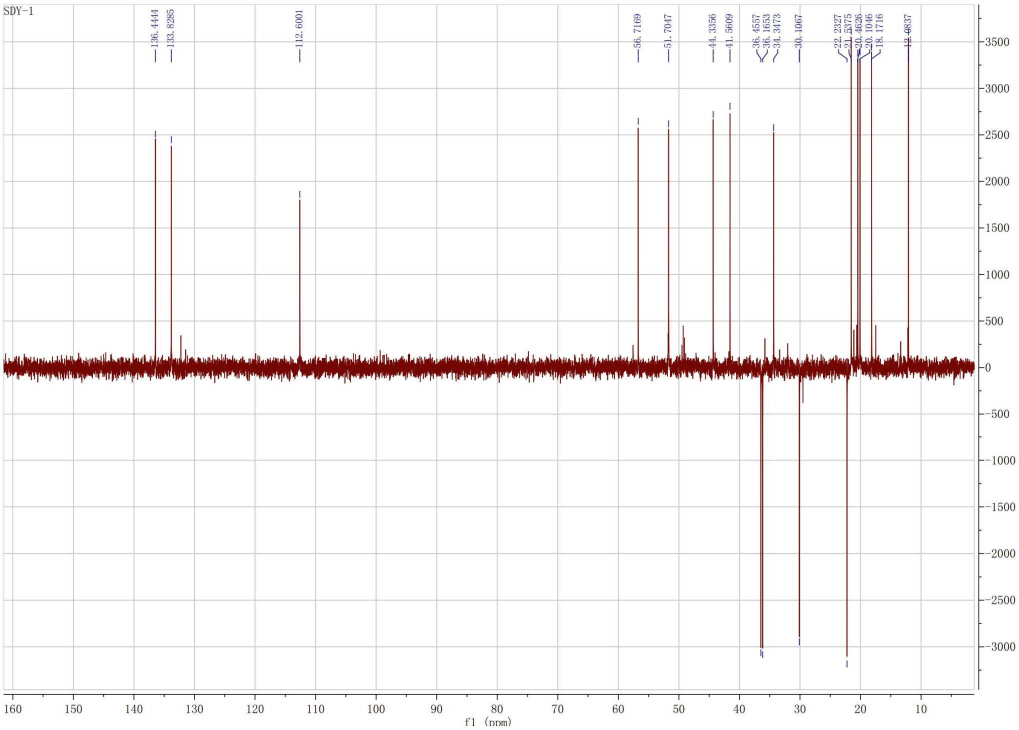

Figure S3. DEPT of Sdy-1

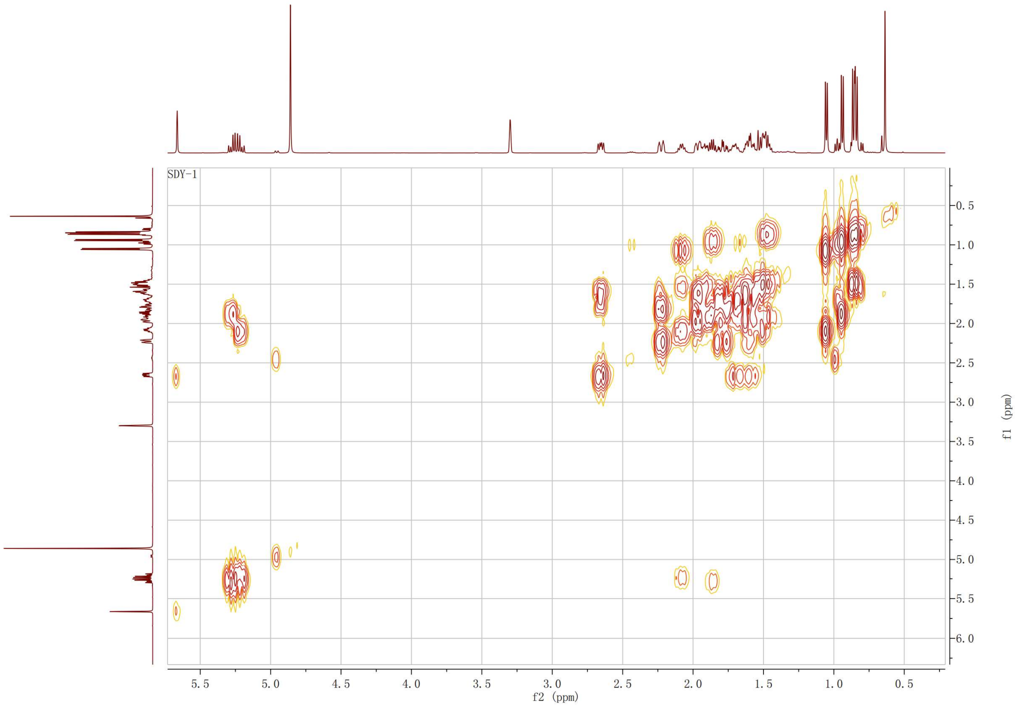

Figure S4. ^{1}H-^{1}H COSY of Sdy-1

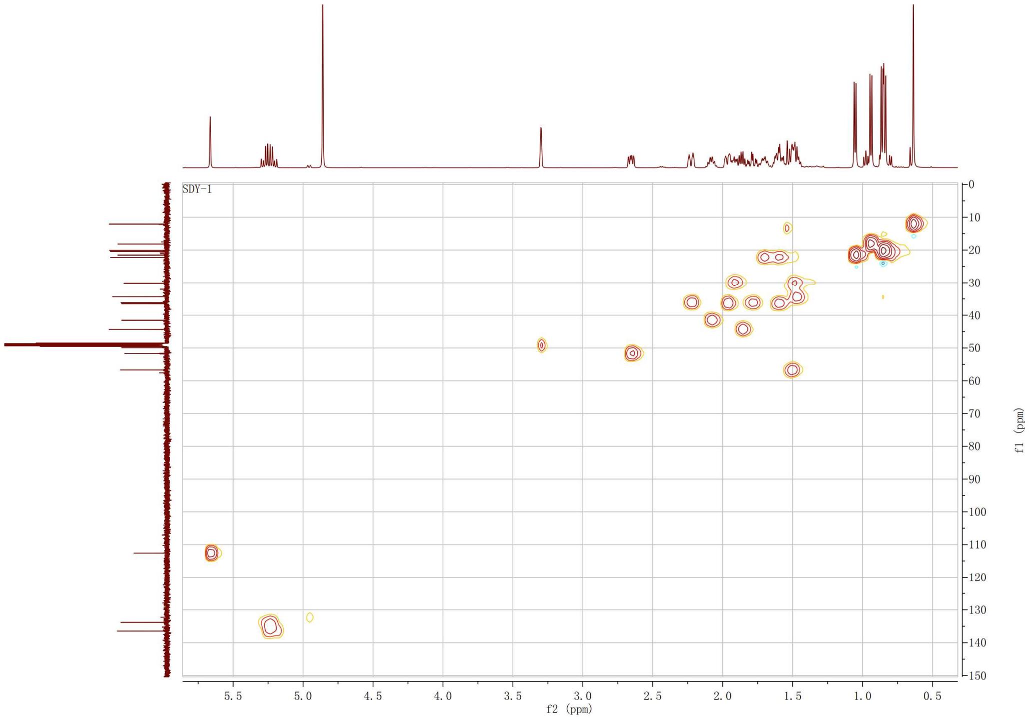

Figure S5. HMQC of Sdy-1

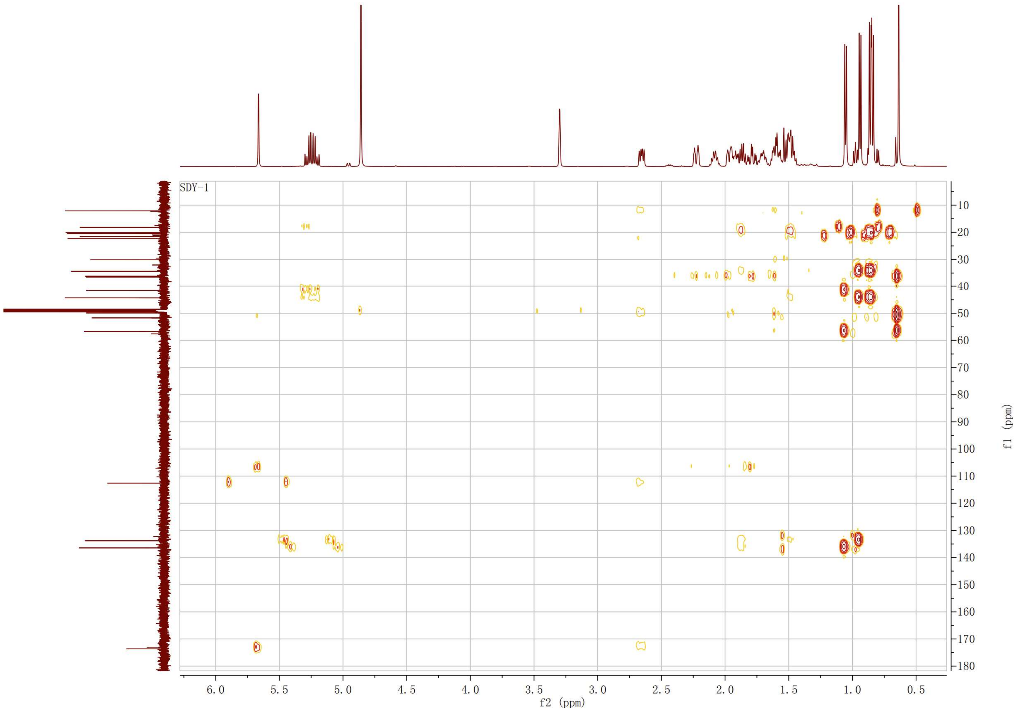

Figure S6. HMBC of Sdy-1

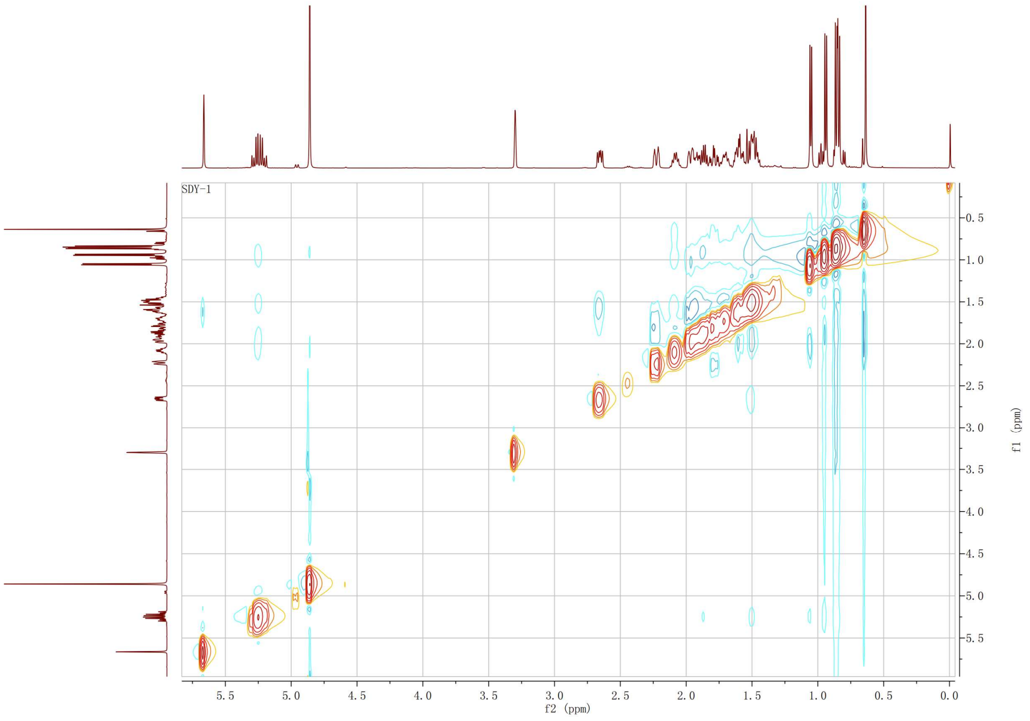

Figure S7. NOESY of Sdy-1

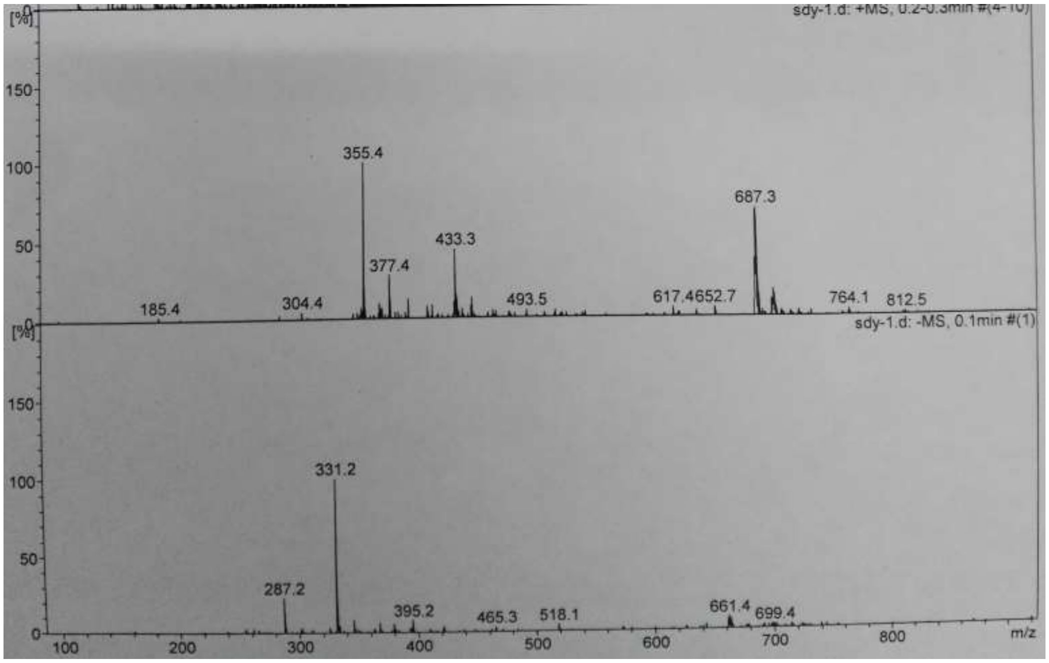

Figure S8. ESI-MS of Sdy-1
